# Task-aware cross-modal refinement and liquid fusion for text-visual grounding

**DOI:** 10.3389/frai.2026.1798247

**Published:** 2026-05-29

**Authors:** Zhirong Li, Changliang Wang, Yongheng Pang, Junchang Xin, Jiaming Zhang, Yunhe Sun

**Affiliations:** 1 Criminal Investigation Police University of China, Shenyang, China; 2 Shanghai Key Laboratory of Crime Scene Evidence, Shanghai, China; 3 Northeastern University, Shenyang, China; 4 Zhejiang University, Hangzhou, China; 5 Shenyang Aerospace University, Shenyang, China

**Keywords:** cross-modal, human-robot interaction, liquid neural networks, multilevel grounding module, visual grounding

## Abstract

**Introduction:**

Visual grounding aims to localize target objects in images based on given textual descriptions, with broad applications in fields such as autonomous driving and human-robot interaction. However, existing visual grounding models still face three major challenges: (1) Most prior works employ separate encoders to process images and text independently, which enlarges the semantic gap between visual and textual features; (2) The use of large-language models leads to excessive parameters, making deployment on lightweight devices difficult; (3) Single-level cross-modal attention mechanisms are insufficient for fully capturing interactive information across modalities.

**Methods:**

To address these issues, this paper proposes a Task-aware Liquid Cross-modal Network (TLCN), which consists of four key modules: a Feature Extraction Module (FEM), a Liquid Fusion Module (LFM), a Task-aware Cross-modal Refinement Module (TCRM), and a Multilevel Grounding Module (MGM). Specifically, the FEM utilizes textual features to guide the extraction of visual features, thereby reducing the feature gap. The LFM employs Liquid Neural Networks (LNNs) to capture temporal dependencies and significantly reduce model parameters. Furthermore, the TCRM deepens textual representation via a second-level attention mechanism, while designed Conv-Trans Blocks (CTBs) are applied to image data to extract deeper visual features. Additionally, a similarity loss function based on KL divergence is introduced to optimize the cross-modal alignment.

**Results:**

The proposed model is extensively evaluated on three widely-used public benchmarks: RefCOCO, RefCOCO+, and RefCOCOg. Moreover, a specialized text localization task is designed for further evaluation. Experimental results demonstrate that the TLCN achieves superior performance across all evaluated datasets and tasks.

**Discussion:**

The superior performance of TLCN validates the effectiveness of its structural designs: text-guided visual extraction successfully bridges the semantic gap, the introduction of LNNs effectively reduces parameter counts for lightweight deployment, and the second-level attention with CTBs sufficiently captures deep cross-modal interactions. These findings suggest that TLCN provides a promising, efficient, and lightweight solution for visual grounding and related localization tasks.

## 1 Introduction

**Figure 1:**
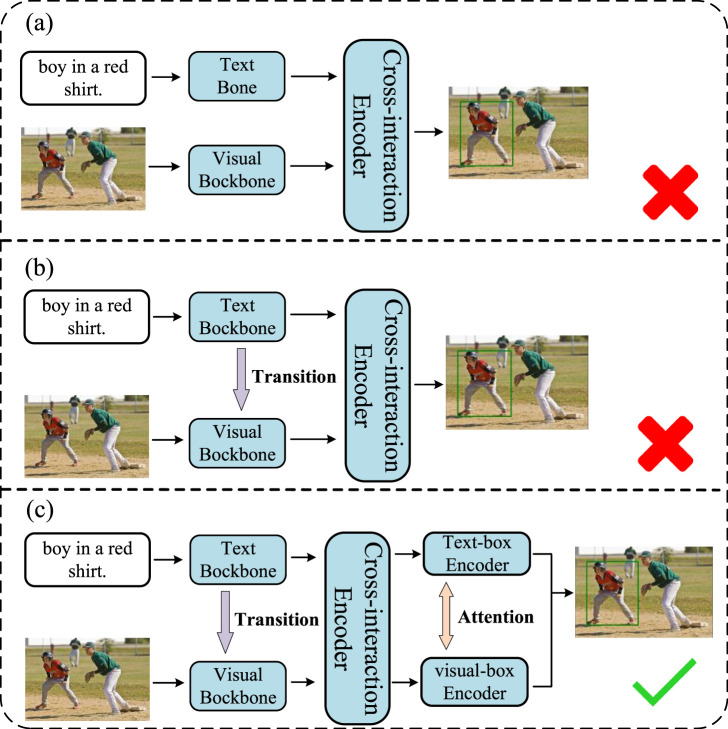
The comparison of visual grounding architectures (images reproduced from the RefCOCO, RefCOCO+, and RefCOCOg datasets, GitHub – lichengunc/refer: Referring Expression Datasets API – GitHub, under Apache 2.0 copyright licensing: http://www.apache.org/licenses/LICENSE-2.0) **(a)** The image and text are encoded using independent encoders, followed by interaction via across-modal attention mechanism. **(b)** During the feature extraction process, textual information is used to modulate the visual features. The two modalities are then fused through cross-modal interaction. **(c)** Textual information is employed to adaptively condition the visual features. After cross-modal interaction, the features are further processed by separate encoders, while explicitly modeling their inter-modal dependencies.

Visual grounding (VG) is a field that integrates image and text modalities for analysis, with broad application prospects in areas such as autonomous driving and robotics (Zhang et al., 2024; Su et al., 2023; Dai et al., 2025; Qiu et al., 2025). It involves localizing a variable number of specific regions in an image based on given textual descriptions. With the advancement of deep learning, the field of computer vision is undergoing exponential transformation (Mane et al., 2025; Huang et al., 2021). In VG, a crucial aspect lies in designing effective image-text fusion models to accurately identify objects present in images. Figure 1 illustrates different modality fusion methods in current VG. In (a), separate encoders are used for images and text, which leads to an excessive semantic gap between visual and textual features. In (b), textual features are used to guide visual feature extraction, followed by a single-level cross-modal interaction; however, this approach struggles to sufficiently capture interactive information between modalities. In (c), textual information is first employed to guide visual feature extraction. Then, following the first-level interaction, the representations of each modality are further refined with explicit consideration of their mutual relationship. Such an architectural design often achieves superior performance.

In deep learning, data, algorithms, and computational power are the three fundamental elements driving continuous evolution. The emergence of large-scale models has provided new perspectives for VG, and leveraging these models for knowledge distillation or comprehensive textual understanding has become a new trend (Yuan et al., 2024). However, although large models can deliver significant performance improvements, their escalating computational demands further constrain their applicability in specific domains. Previous research primarily focused on using large models to understand the fusion of text and images, leading to an exponential increase in required computational resources and posing substantial challenges to model efficiency (Chen et al., 2025). Inspired by the field of biology, *Caenorhabditis elegans* can perform locomotion, motor control, and navigation with only 302 neurons. Building on this, researchers proposed Liquid Neural Networks and applied this architecture to lane-keeping tasks in autonomous driving (Lechner et al., 2020; Hasani et al., 2022). The control network in this model contains 970 times fewer parameters than LSTM and 241 times fewer than CTRNN, achieving superior performance with minimal parameters. It successfully maintains correct lane positioning and demonstrates strong generalization capabilities.

Transformer models have demonstrated powerful performance and achieved remarkable success in various multimodal interaction tasks. Cross-modal transformers are gradually becoming mainstream for interaction in fields such as multimodal emotion recognition and multimodal behavioral analysis (Zhang et al., 2025; Liu et al., 2024; Wang et al., 2024; Li X. C. et al., 2025; Bilotti et al., 2024; Huang et al., 2025). Previous Transformer-based models have attempted to improve image-text interaction, focusing on modality coupling (Huang et al., 2024; Bilotti et al., 2024). However, this approach tends to favor extracting features from one modality, leading to the insufficient representation of the other modality. As a result, the cross-modal interactive features between image and text are not fully captured, causing either textual or visual information to be underrepresented. Moreover, most existing VG models rely on only a single-layer network for detection, which results in a lack of deep hierarchical representations and consequently reduces the model's generalization capability (Ding et al., 2021; Zhu et al., 2026; Zhao et al., 2025).

Therefore, to address the aforementioned challenges, this paper proposes a Task-aware Liquid Cross-modal Network (TLCN). Specifically, to reduce the gap between textual and visual information, we employ textual features to guide the extraction of image features. To alleviate the computational burden caused by large models, we integrate the framework with Liquid Neural Networks (LNNs), applying knowledge distillation to distill knowledge from both video and text modalities into the LNN-based architecture. In order to fully capture cross-modal interaction and further refine each modality after the initial interaction, we design independent encoders for each modality to deepen their respective representations, while explicitly modeling their mutual similarity. For text, we introduce a learnable token to aggregate fused features, which integrates both sentence-level and word-level representations. For video, we propose a Conv-Trans Block (CTBs) to further extract channel-wise contextual information from visual data. Additionally, to enhance the depth information for the grounding task, we incorporate both upsampling and pooling operations, thereby enriching the depth-aware representations for localization. Furthermore, to validate the model's capability in a generalized VG task, we constructed a dataset for text localization, annotating the positions of handwritten text within images. In summary, our contributions can be outlined as follows:

- This paper proposes the Task-aware Liquid Cross-modal Network (TLCN), which incorporates a novel Transformer-based fusion method. Specifically, we introduce a learnable token to simultaneously capture sentence-level and word-level features from the text. For visual representation, we design Conv-Trans Blocks (CTBs) to extract channel-wise information from images.
- The proposed model integrates Liquid Neural Networks (LNNs) to reduce computational requirements, ensuring the model remains lightweight and efficient. Furthermore, we combine upsampling and pooling operations on visual features to enhance depth-aware information for the visual grounding task.
- Extensive experiments were conducted on three benchmark datasets: RefCOCO, RefCOCO+, and RefCOCOg. Furthermore, we designed a generalized visual grounding task for text localization. Experimental results demonstrate the superior performance of the proposed model, with ablation studies confirming the effectiveness of each module.

The structure of this paper is as follows: Section 2 reviews related work in the VG field. Section 3 details the proposed model and its components. Section 4 describes the datasets, training parameters, and experimental setup, and presents the experimental results. Section 5 concludes the paper and discusses future research directions.

## 2 Related work

In this section, we discuss some recent work in visual Grounding, highlight the current limitations in this field, and present the innovations of our approach.

### 2.1 Transformer-based fusion approaches

Given the powerful performance of attention mechanisms in the field of multimodal fusion, an increasing number of researchers are focusing on designing Transformer-based multimodal fusion models (Huang et al., 2024; Wang et al., 2024; Li Z. et al., 2025; Wang et al., 2025). In the domain of multimodal sentiment analysis, (Zhang et al. 2026) proposed the Two-step Hierarchical Attention and Orthogonal Information Enhancement Model (TA-OEM), which employs a two-level attention mechanism to more fully explore fine-grained interactions among features and reduce noise effects in audio-visual data. (Sun et al. 2025) introduced the Conv-Enhanced Transformer and Robust Optimization Network (CTRN), which sequentially computes attention across different features using the CTRN structure to achieve multimodal attention coupling. The approach also integrates Auxiliary Robust Optimization (ARO), where adversarial training is applied to prevent the loss of intra-modal information. Considering the relevance between text and images, and aiming to reduce excessive redundant parameters.

In VG, images often contain a large amount of superficial information that contributes minimally to reasoning about target objects, leading to confusion in target features. To mitigate redundancy, (Zhao et al. 2025) proposed the Context-driven Sparse Decoding Network (CSDNet), which achieves accurate grounding through multimodal context-aware feature extraction and text-guided sparse reasoning. Specifically, the Text-aware Fusion Module (TFM) reduces target feature confusion, the Context-enhanced Interaction Module (CIM) reconciles discrepancies between remote sensing images and text by modeling multimodal contexts, and the Text-guided Sparse Decoder (TSD) addresses the issue of redundant surface information by performing sparse sampling guided by the text. (Zhu et al. 2026) proposed the Dual Contrastive Alignment Residual Transformer Model (DCART). The model incorporates a Sliding Parallel Residual Transformer Module (SPRT) that splits the standard Transformer encoder into two parts based on a windowing scheme: one part serves as a vision-text feature encoder, while the other is designed as a vision-text decoder through parallel residual connections. The Dual Contrastive Alignment (DCA) module aligns target images with corresponding descriptive texts and detected objects.

### 2.2 Liquid neural networks

Liquid Neural Networks (LNNs) represent a revolutionary artificial intelligence technology, whose core idea is to describe the dynamic process of neuronal state changes in response to inputs using continuous-time differential equations, while allowing time constants to adaptively adjust with inputs (Hasani et al., 2020, 2022). In the current AI landscape dominated by large-scale Transformers, Transformer models have demonstrated outstanding performance in language and vision tasks but are increasingly facing the challenge of the Efficient Compute Frontier (ECF) (Chen et al., 2023; Pawlak et al., 2024). The marginal accuracy gains from additional parameters are diminishing, while computational costs are rising sharply. Furthermore, the rigid structure of traditional networks leads to performance degradation in environments where edge conditions change abruptly. In contrast, LNNs are capable of better capturing signal evolution occurring over continuous time scales. Moreover, their compact network architecture and efficient computational properties offer novel solutions for edge computing tasks (Lechner et al., 2020).

(Hasani et al. 2022) recognized that the expressive power of continuous-time neural networks is constrained by numerical differential equation solvers when deployed on computers. However, obtaining closed-form solutions for given dynamic systems is often infeasible. To address this, they constructed a strictly bounded approximation of the integral solution that emerges in liquid time-constant dynamics. This representation reduces reliance on complex numerical solvers and validates the feasibility of LNNs. (Lechner et al. 2020) applied liquid neural networks to the lane-keeping task in autonomous driving. The model achieved performance comparable to LSTM using only a minimal number of parameters, demonstrating effective lane-keeping behavior. It was further shown that LNNs possess strong generalization capabilities: even with significant input noise, the model could still effectively extract useful information, thereby reinforcing the feasibility and robustness of LNNs in practical applications.

### 2.3 Visual grounding

The objective of VG is to localize a natural number of specific regions in an image based on a given textual description. Since 2021, novel concepts such as grounding-based pre-training, grounding-oriented multimodal large models, generalized visual grounding, and multi-object localization have continuously emerged, bringing forth new challenges accordingly. (Dai et al. 2025) proposed an end-to-end proposal-based framework (PropVG), which seamlessly integrates foreground object candidate generation and applied object understanding without requiring additional detectors. Furthermore, the framework incorporates a Contrastive-based Referent Scoring (CRS) module and a Multi-granularity Target Discrimination (MTD) module, which enhance the model's ability to comprehend and distinguish referred objects and improve recognition capability for missing targets, respectively. Among methods leveraging large models, (Chen et al. 2025) employed LLaMA to perform knowledge distillation between video and text, enabling the model to better understand textual descriptions. However, this approach results in a heavyweight model, posing challenges for deployment efficiency. (Huang et al., 2021) introduced LBYL-Net, which utilizes the relative spatial relationships between target objects and “landmarks” to localize targets. The model incorporates a landmark convolution module to ensure that visual features can be propagated in different directions under the guidance of language descriptions. While this model extracts substantial depth information, it inadequately captures information from individual layers, leading to a lack of single-layer feature representation.

To address the aforementioned issues, this paper proposes the Task-aware Liquid Cross-modal Network (TLCN) from a model lightweighting perspective. Innovatively, TLCN employs Liquid Neural Networks to achieve knowledge distillation between image and text modalities, ensuring model efficiency. Furthermore, TLCN simultaneously considers the feature distinctions between sentence-level and word-level representations in text. Additionally, the detection head designed based on a Feature Pyramid Network (FPN) compensates for missing depth information, effectively enhancing the accuracy of the VG system.

## 3 Proposed model

In this section, we introduce the proposed Task-aware Liquid Cross-modal Network (TLCN), which consists of the following key components: the Feature Extraction Module (FEM), the Liquid Fusion Module (LFM), the Task-aware Cross-modal Refinement Module (TCRM), and the Multilevel Grounding Module (MGM). Each of these modules is described in detail in the subsequent subsections.

### 3.1 Tesk definition

**Figure 2:**
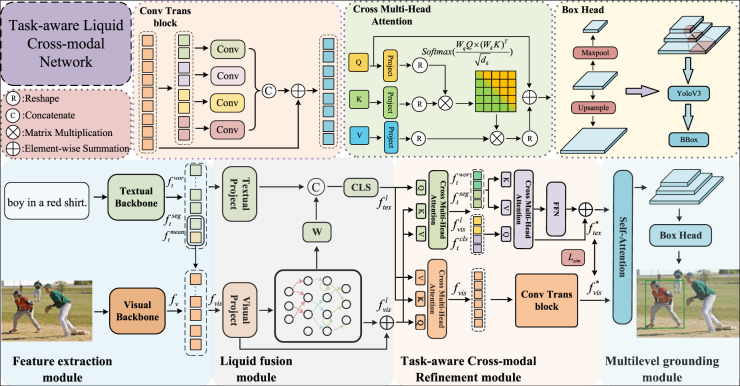
Schematic diagram of the propose Task-aware Liquid Cross-modal Network (images reproduced from the RefCOCO, RefCOCO+, and RefCOCOg datasets, GitHub – lichengunc/refer: Referring Expression Datasets API – GitHub, under Apache 2.0 copyright licensing: http://www.apache.org/licenses/LICENSE-2.0). This model comprises the Feature Extraction Module (FEM), Liquid Fusion Module (LFM),Task-aware Cross-modal Refinement Module (TCRM), and Multilevel Grounding Module (MGM).

In this subsection, we first introduce the VG task. The objective of the VG task is to localize target objects in an image based on textual descriptions. An input sample consists of an image and a corresponding textual description, denoted as *X* ∈ ℝ^c*h*w^ and *T* ∈ ℝ^l*d^, respectively. Where, *c, h, w* represent the number of channels, height, and width of the image, while *L* and *D* denote the sequence length and feature dimension, respectively. The goal of VG is to obtain the localization coordinates *y* = (*x*_*c*_, *y*_*c*_, *h*_*c*_, *w*_*c*_), where are the coordinates of the bounding box center. Thus, the VG model can be formulated as:


$$\begin{array}{l} y=TLCN\left(X,T,\theta_{xt}\right) \end{array}$$ (1)

where θ_*xt*_ represents the parameters of the model. Figure 2 illustrates the overall framework of our proposed model.

### 3.2 Feature extraction module

Traditional feature extraction methods typically employ separate backbones to extract text and video features independently, which can widen the semantic gap between image and text representations. This gap makes it challenging for models to effectively bridge the discrepancies between visual and textual modalities. To address this, we design a novel feature extraction approach that uses textual features to distill knowledge into image features, thereby enabling the image features to incorporate cross-modal semantic information. This allows the model to better understand the complementary information between modalities. Specifically, we first extract visual and textual features using their respective backbones, denoted as *f*_*v*_ and *f*_*t*_. The textual feature *f*_*t*_ comprises three distinct representations, denoted sentence-level features $f_{t}^{seg}\in {\mathbb{R}}^{1*d}$, individual word-level features $f_{t}^{wor}\in {\mathbb{R}}^{l*d}$, and average-pooled word-level features $f_{t}^{mean}\in {\mathbb{R}}^{1*d}$. After extracting the image and text features, we perform knowledge distillation from the text modality to the image modality. In particular, we designed feature $f_{t}^{w}$:


$$\begin{array}{l} f_{txt}^{w}=\alpha_{seg}\phi \left(f_{t}^{seg}\right)+\alpha_{mean}\phi \left(f_{t}^{mean}\right) \end{array}$$ (2)

In the equation, ϕ(·) denotes a single-layer Feed-Forward Networks (FFN), while both α_*seg*_ and α_*mean*_ represent learnable parameters. Subsequently, based on $f_{t}^{w}$, we define the scaling parameter $\gamma =\phi \left(f_{txt}^{w}\right)+\gamma^{learn}$ and the shift parameter $\beta =\phi \left(f_{txt}^{w}\right)+\beta^{learn}$. Given the visual features *f*_*v*_ which are the output of the i-th transformer block layer, we compute its mean μ(*f*_*v*_) and standard deviation σ(*f*_*v*_). The visual features are then modulated by the corresponding scaling and shifting coefficients as:


$$\begin{array}{l} f_{vis}=\gamma \left(\frac{f_{v}-\mu \left(f_{v}\right)}{\sigma \left(f_{v}\right)+\varepsilon }\right)+\beta \end{array}$$ (3)

where ε is a small constant introduced to prevent division by zero. Based on this, we obtain the distilled visual feature *f*_*vis*_.

### 3.3 Liquid fusion module

**Figure 3:**
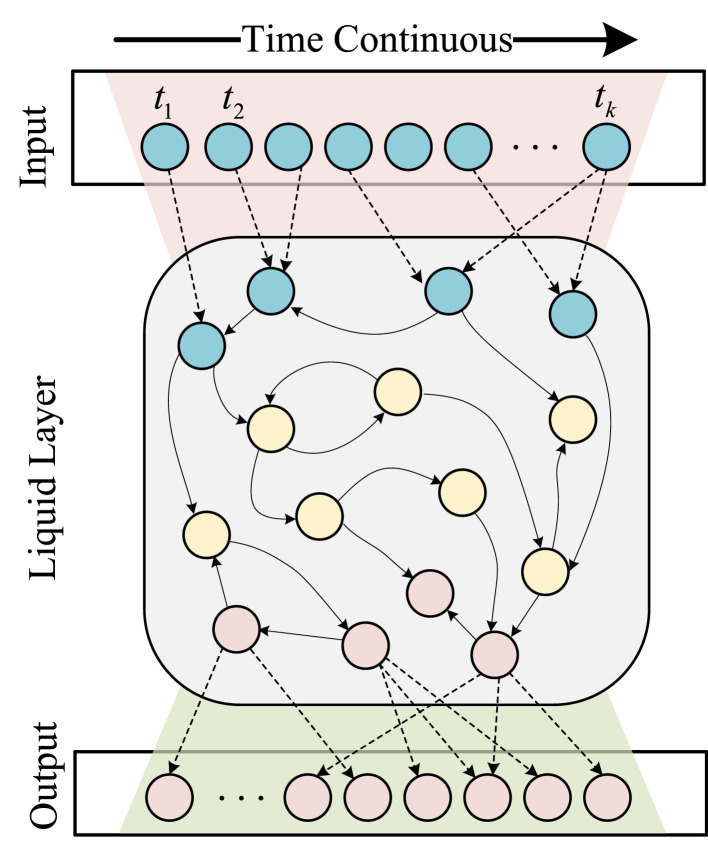
Schematic diagram of the Liquid Fusion Module.

Large language models (LLMs) are widely used due to their excellent text comprehension capabilities; however, they also impose significant performance demands. Previous work has utilized LLMs to extract deeper textual features and achieved promising results, yet such approaches are computationally demanding. The substantial size of these models makes them difficult to deploy on small-scale devices. To address the issue of model parameters, we employs small-parameter Liquid Neural Networks (LNNs) to perform deep extraction of image-text pair information. Specifically, the feature *f*_*vis*_ obtained after FEM processing is first projected via a Visual Projector and then processed using LNNs. The detailed process is illustrated in Figure 3. Following the model proposed by Lechner et al., we apply liquid time-constants (LTC) for processing. The detailed parameter update method for LNNs can be referred to in the study by Lechner et al. To mitigate the gradient vanishing problem that often occurs during LNN training, we incorporate residual connections. This module can be expressed as:


$$\begin{array}{l} f_{vis}^{l}=Project\left(f_{vis}\right)+NCPs\left(Project\left(f_{vis}\right)\right) \end{array}$$ (4)

The LNNs we employ require only half the parameters of a FFN while achieving superior comprehension of sequential data compared to the FFN. This approach allows us to effectively control the model's parameter scale, ensuring its feasibility for deployment on small-scale devices. Furthermore, the features extracted by LNNs are concatenated with textual features to enhance the representation of visual information within the textual modality, while incorporating a learnable CLS token. The specific procedure can be formulated as follows:


$$\begin{array}{l} f_{tex}^{l}=\left[f_{t}^{cls},W_{l}\left(f_{lnn}\right),f_{t}^{seg},f_{t}^{wor}\right] \end{array}$$ (5)

where, *f*_*lnn*_ and *W*_*l*_ denote the features obtained from the LNNs and the projection layer, respectively.

### 3.4 Task-aware cross-modal refinement module

Upon obtaining the textual features $f_{tex}^{l}$ and visual features $f_{vis}^{l}$, most prior research employs cross-modal attention mechanisms to achieve feature fusion. However, relying solely on this mode of interaction can introduce excessive redundant information, which acts as noise that interferes with the exchange of relevant signals. To address this issue, we design a second-level attention mechanism for the textual modality to extract more essential feature information from the text. For the visual modality, we propose a Conv-Trans Block to capture feature information across different channels of the image.

Specifically, we first apply the first-level attention mechanism to enable interaction between image and text (see Figure 2 for details). First, a projection layer maps both the image and text features into a fixed-dimensional space. These two sets of features are then fused via cross-attention, where the Query from one modality is computed with the Key and Value from the other to derive attention scores between the features. Taking the text modality as an example, the first-level attention can be formulated as:


$$\begin{array}{l} Q_{tv}=linear\left(f_{tex}^{l}\right)\\ K_{tv}=linear\left(f_{vis}^{l}\right)\\ V_{tv}=linear\left(f_{vis}^{l}\right) \end{array}$$ (6)

$$\begin{array}{l} X_{tv}=Softmax\left(\frac{Q_{tv}\cdot K_{tv}^{T}}{\sqrt{d_{tv}}}\right)V_{tv}\in {\mathbb{R}}^{l*d} \end{array}$$ (7)

where, *linear* denotes the projection layer. *Q*_*tv*_ represents the query derived from $f_{tex}^{l}$, while *K*_*tv*_ and *V*_*tv*_ denote the key and value obtained from $f_{vis}^{l}$, respectively. Here, *X*_*tv*_ signifies the features resulting from the first-level attention, and *d*_*tv*_ is a corresponding scaling factor introduced to prevent gradient vanishing caused by the softmax function.

After obtaining the first-level attention score matrix, we perform a second-level attention computation on the text to further extract textual and visual features. This deeper fusion mechanism can more effectively capture the interactive characteristics between text and image. Specifically, we concatenate $f_{t}^{cls}$ and $f_{vis}^{l}$ to form the Query, and concatenate $f_{t}^{seg}$ and $f_{t}^{wor}$ to form the Key and Value, thereby calculating the deep interactive information within the text. The process can be formulated as follows:


$$\begin{array}{l} Q_{tt}=linear\left(\left[f_{t}^{cls},f_{vis}^{l}\right]\right)\\ K_{tt}=linear\left(\left[f_{t}^{seg},f_{t}^{wor}\right]\right)\\ V_{tt}=linear\left(\left[f_{t}^{seg},f_{t}^{wor}\right]\right) \end{array}$$ (8)

$$\begin{array}{l} f_{tex}^{*}=Softmax\left(\frac{Q_{tt}\cdot K_{tt}^{T}}{\sqrt{d_{tt}}}\right)V_{tt}\in {\mathbb{R}}^{l*d} \end{array}$$ (9)

where, $f_{tex}^{*}$ represents the refined textual features. For the visual modality, we design a Conv-Trans Block (CTB) to process the image features. Specifically, we split the image features into four parts by halving both the channel count and the feature dimension. Each part is then processed using convolution. The processed features are subsequently concatenated to restore the original shape, followed by a residual connection to prevent gradient vanishing.

Treating the visual and textual modalities with separate encoders, while effective for extracting modality-specific information, can lead to an excessive gap in cross-modal interaction. To mitigate this issue, we design a similarity loss function to reduce the discrepancy between the modalities. Notably, as these features are represented as vectors, we can model their underlying distributions. Given a sufficiently large dataset, we assume both modalities follow a normal distribution $x_{k}\sim N\left(x_{k};\mu_{k},\sigma_{k}^{2}\right)$. We employ KL-divergence to measure the similarity between the visual and textual feature distributions. Let *P*_*tv*_ and *P*_*vt*_ represent the distributions of features $f_{tex}^{*}$ and $f_{vis}^{*}$, where *t* and *v* denote textual and visual modalities. Thus, the similarity loss function can be calculated as:


$$\begin{array}{l} L_{sim}=D_{kl}\left(P_{tv}||P_{vt}\right)+D_{kl}\left(P_{vt}||P_{tv}\right) \end{array}$$ (10)

In the formula, *D*_*kl*_(·) represents the KL-divergence, and *L*_*sim*_ denotes the resulting similarity loss. A smaller value of this loss indicates a higher degree of similarity between the two feature distributions.

### 3.5 Multilevel grounding module

To enhance the depth of features, we perform upsampling and pooling operations on the final feature maps before feeding them into the localization module. We adopt an anchor-based bounding box regression head from YOLOv3 as the detection head. The final output dimension is *K*×*A*, where *A* = 3 denotes the number of anchors and *K* = 5 corresponds to the parameters (*x, y, w, h, s*). The first four values represent the bounding box offsets relative to the predefined anchor boxes, while the last one is the confidence score indicating whether an object exists at that location. Following [1], only the anchor with the largest IoU with the ground-truth bounding box is assigned as a positive sample, and the rest are treated as negative samples. Since this task aims to localize only the single object referred to by the sentence, we maintain exactly one positive sample. For the ranking loss, we optimize by maximizing the distance between the positive sample and the negative samples, which is implemented using a cross-entropy loss. Additionally, for bounding box regression, we employ a combination of Smooth L1 loss and GIoU loss. Therefore, the overall loss function is defined as:


$$\begin{array}{l} L=L_{smooth-l1}+L_{GIoU}+\alpha *L_{CE}+\beta *L_{sim} \end{array}$$ (11)

where *L*_*smooth*−*l*1_, *L*_*GIoU*_, *L*_*CE*_, *L*_*sim*_ denote the Smooth L1 loss, GIoU loss, cross-entropy loss, and similarity loss, respectively. Here, α and β are the weighting coefficients.

## 4 Experiment result

In this section, we will describe the selected datasets, the evaluation metrics adopted, as well as the training details and validation results. Ablation experiments are conducted on each module in the proposed TLCN to verify the effectiveness of individual components. Additionally, we validate the model's generalization capability through text localization tasks in the broader context of generalized VG.

### 4.1 Datasets

In this experiment, we employ three classic datasets in visual grounding: RefCOCO (Yu et al., 2016), RefCOCO+ (Yu et al., 2016), and RefCOCOg (Mao et al., 2015).

**RefCOCO**: This dataset is constructed from images selected from MS-COCO and collected via a two-player game. It comprises 19,994 images, 142,209 referring expressions, and 50,000 object instances. The dataset is partitioned into a training set, a validation set, Test Set A, and Test Set B. Test Set A contains images with multiple people, while Test Set B consists of images with multiple instances from other object categories. This dataset focuses more on basic referring expressions in everyday scenes.

**RefCOCO+**: Similarly derived from MS-COCO images, this dataset contains 19,992 images, 141,564 referring expressions, and 49,856 object instances. It is also divided into a training set, a validation set, Test Set A, and Test Set B. Unlike RefCOCO, the referring expressions in this dataset prohibit the use of absolute location words, forcing models to rely more on understanding visual attributes and relative spatial relationships.

**RefCOCOg**: This dataset consists of 26,711 images, 85,474 referring expressions, and 54,822 object instances. It features longer and more complex descriptive sentences, posing greater challenges to a model's semantic parsing and contextual reasoning capabilities. The dataset has two partitioning schemes: UMD and Google. Following prior work (Dai et al., 2025), we adopt the UMD split, which divides the dataset into training, validation, and test sets.

### 4.2 Experimental settings

The proposed TLCN model was implemented using the PyTorch deep learning framework. Training and testing on the aforementioned datasets were conducted on an NVIDIA GeForce RTX 3090 GPU. For the text backbone, the T5 model was employed, while the Swin Transformer served as the image backbone. Both the text and image features had a dimensionality of 768. Detailed model parameters are presented in Table 1. The weight coefficients A and B were selected from the set {0.2, 0.4, 0.6, 0.8, 1.0} and were ultimately determined to be {0.4, 0.6} based on experimental validation. The model is trained for 100 epochs with a random seed of 123. To stabilize training and mitigate overfitting, we incorporated a learning rate warmup strategy and an early stopping mechanism. Furthermore, various data augmentation techniques were applied to the input images, including rotation, flipping, random cropping, and color jittering.

**Table 1:** Experimental parameter settings for RefCOCO, RefCOCO+, and RefCOCOg datasets.

Parameter	RefCOCO	RefCOCO+	RefCOCOg	Our-texts
Batch size	64	64	128	16
Learning rate	1e-4	1e-4	5e-4	1e-5
Learning rate of T5	1e-4	1e-4	1e-4	1e-4
Optimizer	AdamW	AdamW	AdamW	AdamW
Weight decay	1e-4	1e-4	1e-4	1e-6
Hidden dim	256	256	512	64
Heads nums	8	8	8	4
d-ff in FFN	128	128	256	32
Loss weight α	0.4	0.4	0.4	0.4
Loss weight β	0.6	0.6	0.6	0.6

The evaluation metrics employed in our study align with those used in prior works. Specifically, a prediction for a referred object is considered correct if the Intersection over Union (IoU) between the predicted bounding box and the ground-truth box exceeds 0.5, denoted as Pr@0.5. We report the model's accuracy on each dataset. Furthermore, in Section 4.3, we also provide a more comprehensive evaluation by reporting results from Pr@0.5 to Pr@0.9 with a step size of 0.1.

### 4.3 Results and analysis

#### 4.3.1 Quantitative results

We report the experimental results on the RefCOCO, RefCOCO+, and RefCOCOg datasets, as detailed in Table 2. Initially, we compared TLCN with models that employ traditional architectures, specifically VGG16 and LSTM as the visual and textual backbones, respectively (e.g., CMN, LGRAN). TLCN outperforms these models across all metrics, suggesting that the Transformer-based pre-trained backbone is capable of extracting finer-grained feature information compared to conventional convolution-based feature extraction networks. Furthermore, a comparison between SQC-Base and NMTree reveals that the BERT-based textual backbone achieves an improvement of approximately 3 percentage points over LSTM, underscoring the advantage of modern language models.

**Table 2:** The experimental results of TLCN and other baselines on the RefCOCO, RefCOCO+, and RefCOCOg datasets.

Model	Visual backbone	Textual backbone	RefCOCO			RefCOCO+			RefCOCOg	
			val	testA	testB	val	testA	testB	val	Test
CMN (Hu et al., 2016)	VGG16	LSTM	-	71.03	65.77	-	54.32	47.76	-	-
LGRAN (Wang et al., 2019)	VGG16	LSTM	-	76.60	66.40	-	64.00	53.40	61.78	-
TransVG (Deng et al., 2021)	ResNet50	BERT	80.49	83.28	75.24	66.39	70.55	57.66	67.93	67.44
SQC-Base (Yang et al., 2020)	ResNet101	LSTM	-	78.42	65.53	-	69.07	51.99	-	-
NMTree (Liu et al., 2019)	ResNet101	BERT	76.41	81.21	70.09	66.46	72.02	57.52	65.87	66.44
CMI (Li et al., 2023)	ResNet101	BERT	81.92	83.40	77.37	68.49	72.18	60.30	69.08	69.04
Word2Pix (Zhao et al., 2024)	ResNet101	BERT	81.20	84.39	78.12	69.74	76.11	61.24	71.34	-
MCN (Luo et al., 2020)	DarkNet53	GRU	80.08	82.29	74.98	67.16	72.86	57.31	66.46	66.01
LBYL-Net (Huang et al., 2021)	DarkNet53	BERT	79.67	82.91	74.15	68.64	73.38	59.49	62.70	-
SeqTR (Zhu et al., 2022)	DarkNet53	GRU	81.23	85.00	76.08	68.82	75.37	58.78	71.35	71.58
TransVG++ (Deng et al., 2023)	ViT-S	BERT	82.93	85.45	77.67	69.17	74.46	59.59	70.98	71.83
QRNet (Ye et al., 2022)	Swin-S	BERT	84.01	**85.85**	**82.34**	72.94	76.17	63.81	**73.03**	72.52
TLCN (ours)	ResNet101	T5	81.09	83.41	75.75	68.63	72.95	57.92	66.35	65.91
TLCN (ours)	Swin-B	T5	**84.91**	85.68	81.36	**73.47**	**78.96**	**63.99**	71.59	**73.45**

The bold values indicate the highest accuracy.

Subsequently, we contrasted TLCN with models utilizing ResNet101 and BERT as their visual and textual backbones (e.g., NMTree, CMI, and Word2Pix). TLCN surpasses these models on all metrics, demonstrating its superiority. Notably, the Word2Pix model exhibits a significant discrepancy between its two metrics on the RefCOCO+ dataset, indicating a potential overfitting issue on this specific dataset. This suggests that the model achieves performance gains at the expense of robustness. In contrast, TLCN achieves consistent results across the metrics on this dataset, highlighting its enhanced robustness. We also compared TLCN with models employing DarkNet53 and GRU as the visual and textual backbones (e.g., MCN and SeqTR). TLCN achieved the optimal performance. Although SeqTR's performance on the RefCOCO testA metric is less than 1 percentage point lower than TLCN, its performance on the other two metrics shows a significant drop, which further indicates the superior stability of TLCN.

Finally, we compared TLCN with models that also use a Transformer-based backbone (e.g., TransVG++ and QRNet). TLCN achieved the best performance on the majority of metrics. Specifically, compared to QRNet, the performance difference on the RefCOCO testA and testB metrics is only 0.2 and 1 percentage point, respectively. However, with the assistance of LNNs, TLCN's model parameters are significantly lower than those of the aforementioned models. This indicates that TLCN can be deployed on small-scale devices while maintaining comparable performance, further demonstrating its superiority and portability.

#### 4.3.2 Ablation study

In Table 3, we present the results of the ablation study conducted by systematically removing individual modules from the full TLCN architecture to evaluate their respective contributions to the overall performance. The Baseline configuration represents the model with all proposed modules removed. Specifically, “W/o Swin” denotes the replacement of the Swin Transformer visual backbone with ResNet101, “W/o LFN” indicates the substitution of the LFN with a standard fully connected layer, and “W/o MGM” signifies the removal of the maximum pooling and upsampling operations.

**Table 3:** Ablation experimental results table on the RefCOCO dataset, where “W/o” denotes the removal of the corresponding module.

Model	RefCOCO		
	val	testA	testB
Baseline	66.90	69.31	63.20
W/o Swin	81.09	83.41	75.75
W/o LFN	83.42	84.80	80.56
W/o TCRM	79.31	82.20	77.72
W/o MGM	82.35	83.10	78.24
W/o CTBs	82.76	84.47	79.09
TLCN(ours)	**84.91**	**85.68**	**81.36**

Specifically, “W/o Swin” denotes the configuration where the Swin-B visual backbone is replaced by ResNet101. The bold values indicate the highest accuracy.

Our initial observation is that the removal of any single module consistently leads to a degradation in model performance. The most significant performance drops are observed upon the removal of the TCRM and the Swin backbone. This highlights the substantial performance gain provided by the second-level attention calculation applied to the textual features. The second level of attention effectively extracts additional information not fully captured by the first level, thereby promoting a deeper image-text interaction and ensuring information completeness.

The removal of the LFN module results in a performance decrease of 1 percentage point. Although the impact of LFN on absolute performance is relatively minor, the introduction of LNNs allows the model to more thoroughly understand the image-text interaction with only a marginal increase in parameter count. When compared to models like Word2Pix (Zhao et al., 2024), the inclusion of LNNs contributes to superior robustness, thus enhancing the model's overall stability.

The absence of the MGM module leads to a 2 percentage point performance drop. This demonstrates that the embedding provided by MGM enables the model to acquire deeper image information, which is crucial for performance improvement. Similarly, the removal of the CTBs also results in a 2 percentage point performance reduction. This suggests that the split convolution operation within CTBs allows the model to more comprehensively capture object-level information within the image, thereby boosting performance.

Notably, when all proposed modules are removed (i.e., the Baseline), the model's performance drops by approximately 15 percentage points. This substantial decline conclusively validates the effectiveness and necessity of each proposed module in enhancing the model's overall performance.

Table 4 presents a comparison of the model's accuracy across different values of the weighting factor β for the similarity loss *L*_*sim*_, where β is varied within the set {0, 0.2, 0.4, 0.6, 0.8, 1.0}. As shown in the table, the TLCN model achieves its highest accuracy when the value of β is set to 0.4. Furthermore, we observe a consistent decline in model performance as the value of β increases beyond this optimum. This trend suggests that an excessively high weight assigned to the similarity loss (*L*_*sim*_) can lead to an increased bias in the learned features within the model, thereby hindering overall performance. This analysis confirms the importance of selecting an appropriate weight for *L*_*sim*_ to balance the feature learning objectives.

**Table 4:** Experimental results with different values of the parameter β in the TCRM within the RefCOCO dataset.

β	RefCOCO		
	val	testA	testB
0.0	81.23	83.25	77.91
0.2	82.68	83.78	79.37
0.4	**84.91**	**85.68**	**81.36**
0.6	81.96	82.94	78.69
0.8	81.38	82.33	78.15
1.0	80.94	81.83	77.71

The bold values indicate the highest accuracy.

#### 4.3.3 Visualization

**Figure 4:**
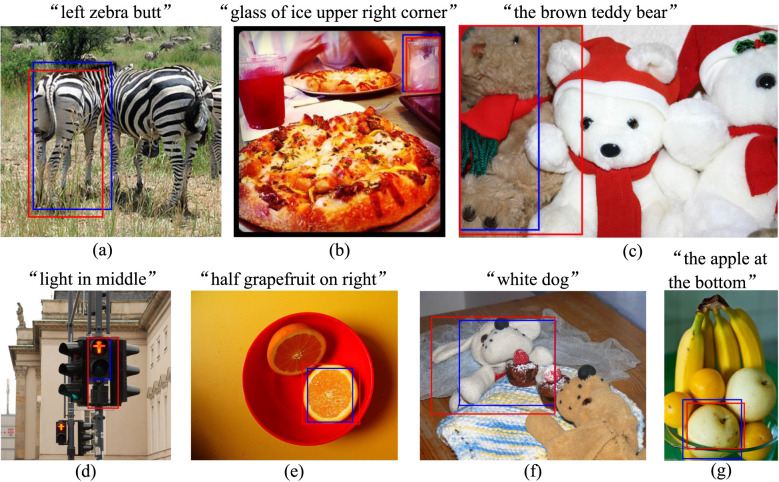
Visualization of localization results of TLCN on the RefCOCOg dataset (images reproduced from the RefCOCOg dataset, GitHub - lichengunc/refer: Referring Expression Datasets API - GitHub, under Apache 2.0 copyright licensing: http://www.apache.org/licenses/LICENSE-2.0). The red bounding boxes represent the ground-truth labels, while the blue bounding boxes denote the model's predictions. **(a)** left zebra butt; **(b)** glass of ice upper right corner; **(c)** the brown teddy bear; **(d)** light in middle; **(e)** half grapefruit on right; **(f)** white dog; **(g)** the apple at the bottom.

To intuitively demonstrate the superior performance of TLCN and enhance its model interpretability, we selected seven representative samples from the RefCOCOg dataset for visual analysis, with the localization results presented in Figure 4.

As shown in Figure 4, TLCN successfully achieves object identification in most cases. However, these results also reveal areas where the model's prediction and reasoning capabilities could be further improved. Specifically, performance degradation is observed in scenarios where the object color is highly similar to the background [Case (f)], and in complex scenes involving severe object occlusion [Case (c) and Case (d)]. In these challenging instances, the model exhibits a deviation in identifying the target object. This limitation may stem from the fact that TLCN did not employ sophisticated color processing during training and failed to fully comprehend the complex scene graph in these scenarios.

In summary, these qualitative visualization results not only validate the superior understanding and reasoning capabilities of our proposed method in the VG task but also showcase the generalization capability and interpretability of TLCN in visual-language tasks.

#### 4.3.4 Case study

##### 4.3.4.1 Model generalization and text localization on handwritten signatures

**Figure 5:**
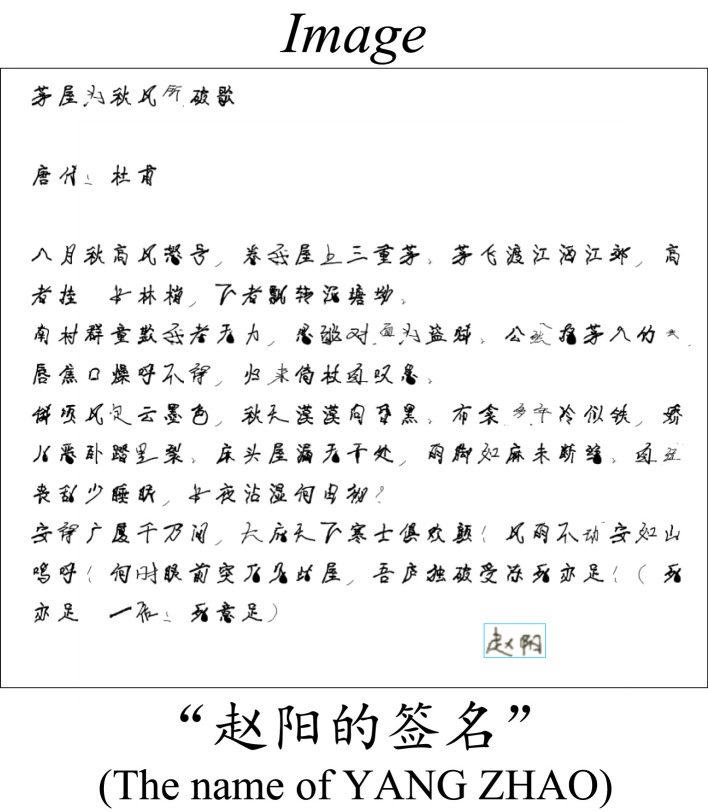
This is an example from our custom text localization dataset. The blue bounding box indicates the ground-truth area for text localization, with the text label provided in English within parentheses.

To evaluate the model's generalization capability, we designed a text localization task utilizing a custom handwritten signature dataset. Structured consistently with typical visual localization tasks, this dataset comprises 300 samples, where each sample includes an image of a signature and a corresponding descriptive text. Further details on the dataset structure are provided in Figure 5.

**Figure 6:**
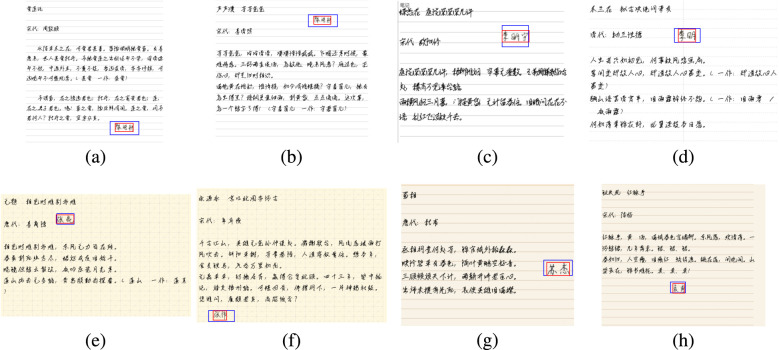
Visualization of text localization results of TLCN on the handwritten signature dataset. The red bounding boxes represent the ground-truth labels, while the blue bounding boxes denote the model's predictions. **(a)** The name of Haoxuan Chen; **(b)** The name of Haoxuan Chen; **(c)** The name of Mingyu Li; **(d)** The name of Ming Li; **(e)** The name of Liang Zhang; **(f)** The name of Wei Zhang; **(g)** The name of Jie Su; **(h)** The name of Yong Yuan.

The dataset was randomly partitioned into an 80% training set and a 20% test set. The model was trained using the parameters specified in Table 1. To ensure robustness, the experiment was repeated three times, and the final results were reported as the average of these runs. The results demonstrate a Pr@0.5 accuracy of 69.62%. Figure 6 visualizes the model's localization performance on selected samples. The visualization confirms that the model successfully localizes the text, suggesting promising performance for the downstream task of person-specific handwritten signature recognition.

## 5 Conclusion

This paper proposes the Task-aware Liquid Cross-modal Network (TLCN), which effectively integrates image and text modalities to address the VG task. The TLCN architecture is primarily composed of four distinct modules: the Feature Extraction Module (FEM), the Liquid Fusion Module (LFN), the Task-aware Cross-modal Refinement Module (TCRM), and the Multilevel Grounding Module (MGM). The FEM utilizes textual features to guide the extraction of image features, thereby mitigating the feature gap between the two modalities. The LFN employs Liquid Neural Networks (LNNs) to process the data, which not only captures temporal dynamics but also significantly reduces the model's parameter count. The TCRM deepens the textual feature representation using a second-level attention mechanism and extracts deep visual features via a specially designed Conv Trans Block (CTB). Within this module, a similarity loss function based on Kullback-Leibler (KL) divergence is also introduced. The MGM provides the model with deep hierarchical information from the image. Detailed comparisons against other baseline models on three benchmark visual localization datasets demonstrate that TLCN achieves superior performance across all metrics. Ablation studies confirm the effectiveness and necessity of each proposed module. Furthermore, to validate the model's generalization capability, we designed a text localization task, a variant of the super-visual grounding task. Experimental results show that TLCN can accurately locate the specific position of the target text, indicating promising performance for the downstream task of person-specific handwritten signature recognition.

## Data Availability

The raw data supporting the conclusions of this article will be made available by the authors, without undue reservation.
